# A feedback loop between GATA2-AS1 and GATA2 promotes colorectal cancer cell proliferation, invasion, epithelial-mesenchymal transition and stemness via recruiting DDX3X

**DOI:** 10.1186/s12967-022-03483-8

**Published:** 2022-06-25

**Authors:** Yuliang Pan, Yuxing Zhu, Jun Zhang, Long Jin, Peiguo Cao

**Affiliations:** Department of Oncology, the Third Xiangya Hospital of Central South University, No.138, Tongzipo Road, Yuelu District, Changsha, 410013 Hunan China

**Keywords:** Colorectal cancer, GATA2-AS1, GATA2, DDX3X

## Abstract

**Background:**

Colorectal cancer (CRC) is a common malignant tumor with a high risk of metastasis. Long non-coding RNAs (lncRNAs) have been reported to be implicated in cancer progression via regulating its nearby gene. Herein, we investigated the function of GATA binding protein 2 (GATA2) and lncRNA GATA2 antisense RNA 1 (GATA2-AS1) in CRC and the mechanism underlying their interaction.

**Methods:**

Colony formation assay, flow cytometry analysis and transwell assay were implemented to detect cell proliferation, apoptosis and invasion. Western blot analysis and sphere formation assay were conducted to assess epithelial-mesenchymal transition (EMT) and cancer stemness of CRC cells. RNA pull down, RNA-binding protein immunoprecipitation (RIP), chromatin immunoprecipitation (ChIP) and luciferase reporter assays were implemented to investigate the regulatory mechanism between GATA2-AS1 and GATA2.

**Results:**

GATA2-AS1 and GATA2 were highly expressed in CRC cells. Knockdown of GATA2-AS1 and GATA2 impeded CRC cell proliferation, invasion, EMT and cancer stemness, and induced cell apoptosis. GATA2-AS1 expression was positively correlated with GATA2. GATA2-AS1 recruited DEAD-box helicase 3 X-linked (DDX3X) to stabilize GATA2 mRNA. GATA2 combined with GATA2-AS1 promoter to enhance GATA2-AS1 expression.

**Conclusion:**

Our study confirmed that a feedback loop between GATA2-AS1 and GATA2 promotes CRC progression, which might offer novel targets for CRC treatment.

**Supplementary Information:**

The online version contains supplementary material available at 10.1186/s12967-022-03483-8.

## Background

Colorectal cancer (CRC) is a common and severe disease which poses a threat to human health [1]. In 2022, there will be approximately 592,232 new cases of CRC in China, 160,248 new cases in the United States and 309,114 deaths from CRC in China, 56,693 deaths in the United States [2]. In patients with early CRC, the five-year survival rate is about 90%. However, this rate declines to < 10% in advanced-stage patients with distant metastases [3]. Great advances have been achieved in early screening and treatment for CRC, but the survival rate of CRC remains unchanged [4]. This disease is associated with low detection efficiency and high death rate due to the lack of symptoms in CRC patients at early stage and metastasis at advanced stage [5]. Metastasis is a process which involves cancer cell invasion, epithelial mesenchymal transition (EMT) and microenvironment changes [6]. Hence, it is vitally important to explore the molecular mechanisms underlying CRC progression.

With the development of next-generation sequencing technologies, long non-coding RNAs (lncRNAs) have been brought into focus [7]. LncRNAs are defined as a type of RNAs longer than 200 nucleotides and incapable of coding proteins [8]. Initially, lncRNA was considered as transcriptional “noise” due to its non-coding characteristic. Nowadays, a growing number of studies have suggested that lncRNA plays a crucial role in a variety of biological processes via regulating gene expression [9]. LncRNAs regulate gene expression in multiple ways: they could impact on transcription by guiding the chromatin-modifying complexes and transcription factors, or by acting as scaffolds of protein–protein interactions; moreover, they could act as microRNA (miRNA) sponges sequestering endogenous miRNAs [10]. Dysregulation of lncRNAs exert tumor-promoting or tumor-suppressing functions to affect the biological processes [11]. Notably, some certain lncRNAs could be considered as potential therapeutic targets or prognostic molecules for cancers [12].

RNA binding proteins (RBPs) are important regulators in many cellular processes including RNA splicing, modifications, localization, stability, degradation as well as translation [13]. They modulate gene expression post-transcriptionally and are implicated in multiple cellular phenotypes [14]. Abnormal expression of RBPs can affect tumorigenesis via controlling RNA or protein homeostasis [15]. Accumulating evidence supports that lncRNAs participate in the development of cancers via interacting with RBPs to mediate the stability of mRNA. A study proposed by Yang Lan and his colleague has manifested that OCC-1 negatively modulates CRC cell growth via destabilization of ELAVL1 protein [16]. Besides, TRPM2-AS facilitates CRC cell proliferation via recruiting TAF15 to regulate the mRNA stability of TRPM2 [17].

Transcription factors are previously defined as “undruggable” targets for the exception of ligand-inducible nuclear receptors [18]. The deeper knowledge of these transcription factors, such as their structure and function including expression, degradation, their ability to interact with co-factors, has changed this hypothesis [19]. Transcription factors are involved in the progression of cancers for their potential oncogenic functions, which paves the way for potential therapies targeted against transcription factors [20].

GATA binding protein 2 (GATA2) and GATA binding protein 2 antisense RNA 1 (GATA2-AS1) have shown to be implicated in tumorigenesis. For instance, GATA2 enhances aggressiveness and resistance to standard therapies against prostate cancer [21]. GATA2-AS1 represses non-small cell lung cancer growth via regulating GATA2 [22]. In this research, we investigated the impacts of GATA2-AS1 and GATA2 on the progression of CRC in vitro and in vivo. Further, we probed into the regulation mechanism between GATA2-AS1 and GATA2 in CRC cells. Our study might provide promising targets for CRC treatment.

## Methods

### Cell lines

CRC cell lines used in this research included DiFi, SW620, DLD-1, HT-29, SW480 and HCT116. DiFi cell line and human normal colonic epithelial cells (HCoEpiC) were supplied by Shanghai Qincheng Biological Technology Co., Ltd. (Shanghai, China); the others were provided by ATCC (Manassas, VA). SW620 and SW480 cell lines were maintained in Leibovitz's L-15 Medium, HT-29 and HCT116 cell lines in McCoy's 5A Medium, and DiFi, DLD-1 and HCoEpiC cells in RPMI-1640 Medium. SW620, HT-29, HCT116 and DLD-1 cells were cultured in the medium containing 10% FBS with the supplementation of 1% penicillin/streptomycin in a humid incubator at 37 °C with 5% CO_2_.

### Quantitative real-time polymerase chain reaction (RT-qPCR)

TRIzol Reagent (Invitrogen; Carlsbad, CA) was used for RNA isolation. Then, the extracted RNA was subjected to reverse transcription for cDNA synthesis using PrimeScript RT Reagent Kit (Takara Bio, Japan). SYBR^®^ Premix Ex Taq™ II (Takara Bio) was used for qPCR. Meanwhile, GAPDH was used as endogenous control. The calculation of gene expression level was based on the 2^−∆∆Ct^ method [23] and experimental data were displayed as mean ± standard deviation (SD). The experiment was repeated three times. Primer sequences were reported in Table 1.

**Table 1:** Primer sequences

Gene	primer sequence
GATA2-AS1	F:CCGGGCAGCTTACGATTCTT R:GCGGTGTCTTTCAGAGGGTC
GATA2	F:AGTCTGTCTATTGCCTGCCGC R: TGCAGACGGCAACGGC
GATA2-AS1 promoter	F:CAACGGGCCCAATTGCC R:ACACGAACCATAGAGCCGAT

F: forward primer; reverse primer

### Cell transfection

For stably silencing GATA2-AS1, GATA2 or DDX3X expression, the specific short hairpin RNAs (shRNAs) to GATA2-AS1 or GATA2 or DDX3X and shRNA of negative control (sh-NC) were designed and supplied by GenePharma (Shanghai, China). The full-length cDNA sequence of GATA2 was inserted into the pcDNA3.1 vectors (Invitrogen), using empty vectors as control. These vectors were transfected into cells for 48 h using Lipofectamine 3000 (Invitrogen).

### Colony formation assay

CRC cells (600 cells each well) were plated in 6-well plates and incubated for 12 days at 37 °C. Subsequently, cells were washed with phosphate buffered saline (PBS), and fixed by 4% paraformaldehyde for 15 min, followed by staining with 0.5% violet crystal for 10 min and colony counting by the manual method. The experiment was performed in triplicate.

### 5-Ethynyl-2’-deoxyuridine (EdU)

CRC cells were plated in 24-well plates. We added 10 μM EdU into each well for incubation. Then, cells were fixed, washed, and added with Click-iT EdU Kit. DAPI was used for nuclear counterstain. Images were observed using a fluorescence microscope (Olympus). The experiment was carried out in triplicate.

### Flow cytometry analysis

Flow cytometry analysis was done for measuring the apoptosis of CRC cells. Transfected cells were collected, washed and then re-suspended in binding buffer (50 mL). Next, cell suspensions were added with staining solution containing Annexin V-FITC (1/500, BioVision, Milpitas, CA) and PI (1/500, Beyotime), followed by analysis utilizing a BD Biosciences FACSCalibur™ Flow Cytometer (San Diego, CA). The experiment was performed in triplicate.

### Terminal-deoxynucleoitidyl transferase mediated nick end labeling (TUNEL)

Transfected cells were washed, fixed and then permeabilized, followed by TUNEL assays utilizing In Situ Cell Death Detection Kit (Roche) based on the supplier’s protocols. Briefly, cells were incubated in terminal dexynucleotidyl transferase (TdT) reaction cocktail (2 μL), and then treated with Click-iT reaction cocktail (50 μL). DAPI (1/2000) was used to counterstain the nuclei. The experiment was performed in triplicate.

Besides, apoptosis of mouse xenografts was evaluated by tissue TUNEL assay, which was performed as per the instructions for the TUNEL assay kit (KeyGen, Nanjing, China).

### Transwell invasion assay

Cells (2 × 10^5^) were put into the upper chamber using an 8-mm pore size of transwell coated with Matrigel (50 μg/well, BD Biosciences). The medium with no serum was supplemented to the upper chamber. Complete medium (500 μL) with 20% FBS was utilized to treat the lower chamber. Subsequent to 24 h of incubation, we slightly wiped the cells on the surface of the upper membrane. The invaded cells into the lower chamber were subjected to 10-min fixation in 4% paraformaldehyde (PFA) and 30-min staining by crystal violet (500 μL). The invaded cells in 5 randomly selected fields were observed and imaged using an inverted microscope. The experiment was performed in triplicate.

### Western blot

Proteins were extracted utilizing RIPA lysis buffer (Thermo Fisher Scientific) and quantified by a BCA Protein Assay Kit (Abcam, Cambridge, MA). Subsequently, 1% SDS-PAGE was utilized for protein separation. Then, protein samples were transferred onto PVDF membranes (Millipore), which were sealed with 5% defatted milk in TBST, followed by overnight incubation with primary antibodies (Abcam) at 4 °C. In this assay, we used primary antibodies as follows: Anti-GATA2 (1/1000), Anti-E-cadherin (1/1000), Anti-N-cadherin (1/1000), Anti-Vimentin (1/1000), Anti-Nanog (1/1000), Anti-OCT4 (1/1000), Anti-DDX3X (1/1000), Anti-Ki-67 (1/1000), Anti-PCNA (1 µg/ml) and Anti-GAPDH (1/1000). Next, the membranes were subjected to 1 h of incubation with secondary antibodies (Abcam) at room temperature. Lastly, protein bands were visualized using ECL western blotting substrate (Invitrogen). The experiment was performed in triplicate.

### Sphere formation

Post transfection, CRC cells were plated on the Corning ultra-low attachment plates (Corning, NY) in medium with no serum, added with 20 ng/mL EGF, 20 ng/mL FGF, 4 mg/mL heparin and 2% B27 (Invitrogen) for 14 days of incubation, followed by analysis of the number and size of spheres. The experiment was performed in triplicate.

### Subcellular fractionation

Nuclear and cytoplasmic fractions of cells were separated using PARIS kit (Life Technologies, Thermo Fisher Scientific) in the light of supplier’s requirements. The expression level of GATA2-AS1 in nuclear or cytoplasmic fraction was determined by RT-qPCR analysis. In this assay, U6 or GAPDH served as a positive control for nuclear/cytoplasmic fraction. The experiment was done in triplicate.

### Fluorescent in situ hybridization (FISH)

To determine the subcellular distribution of GATA2-AS1 in CRC cells, FISH assay was carried out utilizing FISH Tag™ RNA Red Kit (F32952, Invitrogen) as per the user guide. Cells were fixed and washed, followed by incubation with FISH probe specific for GATA2-AS1 (5 μL; RiboBio, Guangzhou, China) in hybridization buffer. Nuclear counterstain was done utilizing DAPI. The slides were observed with a fluorescence microscope. The experiment was done in triplicate.

### Actinomycin D (ActD) assay

ActD (Abcam) at a final concentration of 4 μM was used to treat cells. ActD was added to cells at 0 h, 4 h, or 8 h. RT-qPCR was used to quantify mRNA levels. The experiment was done in triplicate.

### RNA pull down assay

RNA pull down assay was conducted utilizing Pierce Magnetic RNA–Protein Pull-Down Kit (Thermo Fisher Scientific, Waltham, MA) as per the supplier’s protocols. The protein lysates were mixed with GATA2-AS1 or GATA2-AS1 AS or GATA2 or GATA2 AS, followed by addition of magnetic beads. RNA–protein mixture was analyzed using western blot. The experiment was carried out in triplicate.

### RNA-binding protein immunoprecipitation (RIP)

Magna RIP RNA-Binding Protein Immunoprecipitation Kit (Millipore, Bedford, MA) was utilized for RIP conforming to the supplier’s protocols. Cells were subjected to lysis with RIP lysis buffer. Subsequently, cell lysates were subjected to incubation with RIPA buffer containing magnetic beads conjugated with anti-DDX3X antibody or anti-IgG. The RNA extracted from the immunoprecipitates was purified for RT-qPCR analysis. The experiment was implemented in triplicate.

### Chromatin immunoprecipitation (ChIP)

ChIP assay was conducted via a ChIP Assay Kit (Beyotime) following the guidelines of the supplier. Cells were cross-linked with 1% PFA for 10 min and then sonicated into 200–1000 bp fragments. Next, chromatin was immunoprecipitated with anti-GATA2 or IgG antibodies, followed by RT-qPCR. Each experiment was independently performed in triplicate.

### Luciferase reporter assay

The sequence of GATA2-AS1 promoter was amplified from CRC cells. Then, the fragments of Full length, Site 1-MUT, Site 2-MUT and Site 1 + 2 MUT were inserted into the pGL3 luciferase vector (Promega, Madison, WI) to construct reporter vectors. Then, these vectors were co-transfected with pcDNA3.1/GATA2 or sh-GATA2#1/2 into cells. After 48 h of transfection, luciferase activities were determined using Luciferase Assay Kit (Promega). The experiment was performed in triplicate.

### In vivo xenograft experiments

A total of 20 BALB/c nude mice (five-week-old, male) were selected and randomly divided into 4 groups (5 mice for each group), two test groups and two control groups. Each mouse in test groups was injected with stably sh-GATA2-AS1#1-transfected HCT116 or SW480 cells (5 × 10^6^) while same amount of stably sh-NC HCT116 or SW480 cells were injected into each mouse in control groups. We used vernier caliper to measure the volume of xenografts in mice every 4 days. Finally, mice were sacrificed and then tumor tissues were extracted and used for subsequent analyses. Xenografted tumors were weighed by electronic scale. Animal study was approved by the Ethics Committee of the Third Xiangya Hospital of Central South University (2019-S336). Three independent assays were requested.

### Immunohistochemical (IHC) assay

IHC staining was performed on 4-μm thick paraffin-embedded sections. Slides were dewaxed and antigen retrieval, and then incubated with primary antibodies (Abcam) including Anti-Ki-67 (0.1–5 µg/mL), Anti-PCNA (1/10,000–1/30,000), Anti-E-cadherin (1/500), Anti-N-cadherin (1 µg/mL), Anti-Nanog (1/100–1/250) and Anti-OCT4 (1/1000) overnight. Afterwards, secondary antibodies (1/200–1/2000, Abcam) were added for incubation at room temperature. Next, DAB Stain Kit (ZSBio, Beijing, China) was treated into slides and then counterstained by DAPI. The experiment was done in triplicate.

### Bioinformatics analysis

GEPIA (http://gepia2.cancer-pku.cn) was used to analyze GATA2-AS1 expression in colon adenocarcinoma (COAD) samples (n = 275) and normal samples (n = 349). RNA-seq of GATA2-AS1 in 27 different normal tissues was analyzed by NCBI database (https://www.ncbi.nlm.nih.gov). Potential miRNAs of either GATA2-AS1 or GATA2 were predicted by ENCORI (https://rna.sysu.edu.cn/encori/). In addition, UCSC database (http://genome.ucsc.edu/) was used to predict transcription factors of GATA2-AS1. JASPAR (http://jaspar.genereg.net/) was used to predict GATA2-binding sites on the sequence of GATA2-AS1 promoter.

## Statistical analysis

The data were presented as mean ± SD for three independent experiments. GraphPad PRISM 6 was used for statistical analysis. Student’s t-test or analysis of variance (ANOVA) was used for comparison of group differences; Dunnett’s test or Tukey’s test was used following the ANOVA. P value < 0.05 was considered as statistical significance.

## Results

### GATA2-AS1 and GATA2 are highly expressed in CRC cells

**Fig. 1:**
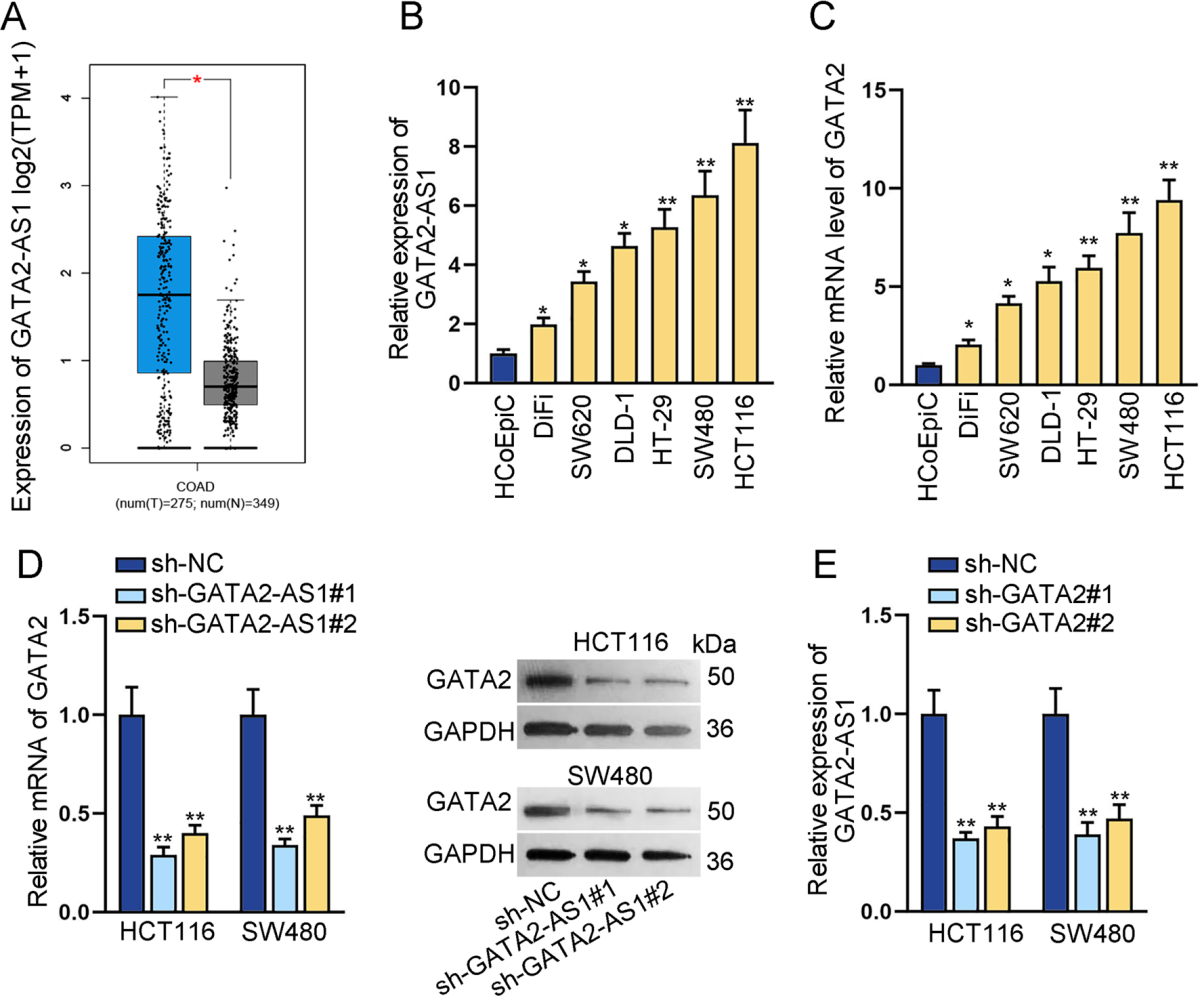
GATA2-AS1 and GATA2 are highly expressed in CRC cells. **A** Box plot showed GATA2-AS1 expression in COAD tissues. **B**, **C** RT-qPCR was applied to analyze the expression of GATA2-AS1/GATA2 in CRC cells. **D** RT-qPCR and western blot were applied for detection of GATA2 RNA and protein levels in GATA2-AS1-deficient CRC cells. **E** GATA2-AS1 expression was detected in GATA2-deleted CRC cells. ^*^P < 0.05, ^**^P < 0.01

To explore the expression profile of GATA2-AS1 in CRC, we used GEPIA2 (http://gepia2.cancer-pku.cn) to analyze GATA2-AS1 expression in colon adenocarcinoma (COAD). Relative to 349 cases of normal tissues, GATA2-AS1 was significantly highly expressed in 275 cases of COAD tissues (Fig. 1A). GATA2-AS1 was lowexpressed in normal colon tissues according to the NCBI database (https://www.ncbi.nlm.nih.gov) (Additional file 1: Fig. S1). The above data confirmed the aberrant elevation of GATA2-AS1 expression in CRC tissues. Moreover, RT-qPCR suggested that GATA2-AS1 expression was markedly increased in six CRC cell lines relative to that in human normal HCoEpiC (Fig. 1B). Intriguingly, we found that the expression of GATA2, nearby gene of GATA2-AS1, was also apparently elevated in CRC cell lines versus that in normal cell line (Fig. 1C). In particular, SW480 and HCT116 cell lines exhibited a relatively higher expression of GATA2-AS1 and GATA2 than other CRC cells did, so they were selected for follow-up experiments.

Besides, it has been suggested that some lncRNAs could modulate the expression of nearby genes [24]. Then, we investigated the regulatory relationship of GATA2-AS1 and GATA2 in CRC cells. Before that, two specific shRNAs for GATA2-AS1 were transfected into SW480 and HCT116 cells to silence GATA2-AS1 expression (Additional file 2: Fig. S2A). GATA2 mRNA and protein levels were overtly reduced when GATA2-AS1 was knocked down (Fig. 1D). Likewise, we also silenced GATA2 expression via transfection of sh-GATA2 plasmids (Additional file 2: Fig. S2B). It was found that GATA2 depletion obviously decreased the expression of GATA2-AS1 (Fig. 1E). These results suggested that GATA2-AS1 and GATA2 are expressed in CRC cells at high level and that their both expression is tightly regulated.

### GATA2-AS1 and GATA2 promote CRC cell proliferation while represses cell apoptosis

**Fig. 2:**
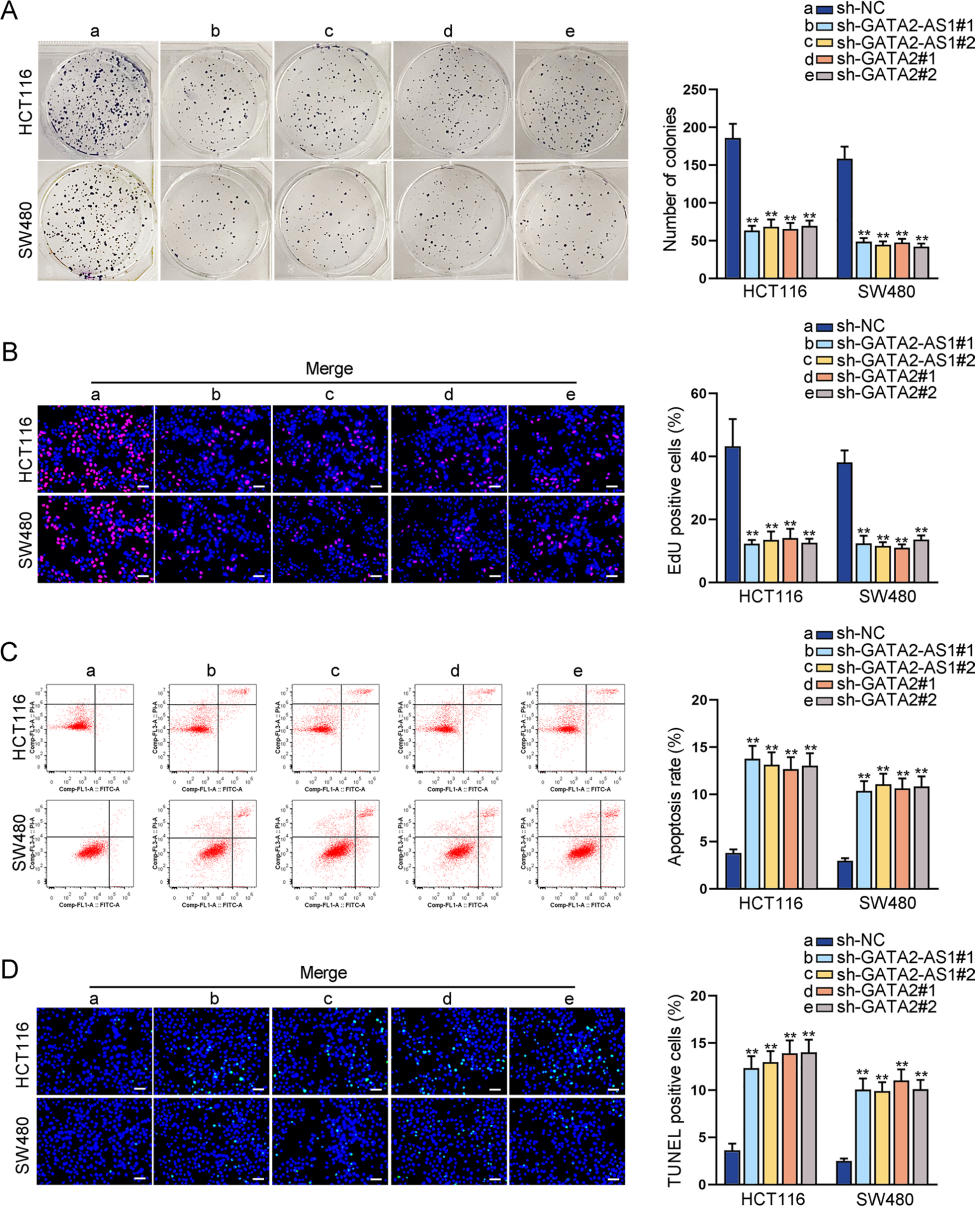
GATA2-AS1 and GATA2 promote CRC cell proliferation and repress cell apoptosis. **A**, **B** Colony formation and EdU assays were carried out to assess the impacts of GATA2-AS1 or GATA2 silencing on CRC cell proliferation. **C**, **D** Apoptosis assays were used to analyze the impacts of GATA2-AS1 or GATA2 deficiency on CRC cell apoptosis. ^**^P < 0.01

Next, we explored the impact of GATA2-AS1 or GATA2 on CRC cell proliferation and apoptosis. It was found that deficiency of GATA2-AS1 or GATA2 markedly hampered the proliferation capacity of CRC cells (Fig. 2A, B). The apoptosis of CRC cells was elevated in GATA2-AS1-silenced or GATA2-silenced SW480 and HCT116 cells (Fig. 2C, D). Altogether, both GATA2-AS1 and GATA2 promote CRC cell proliferation and repress cell apoptosis.

### GATA2-AS1 and GATA2 promote CRC cell invasion, EMT and stemness

**Fig. 3:**
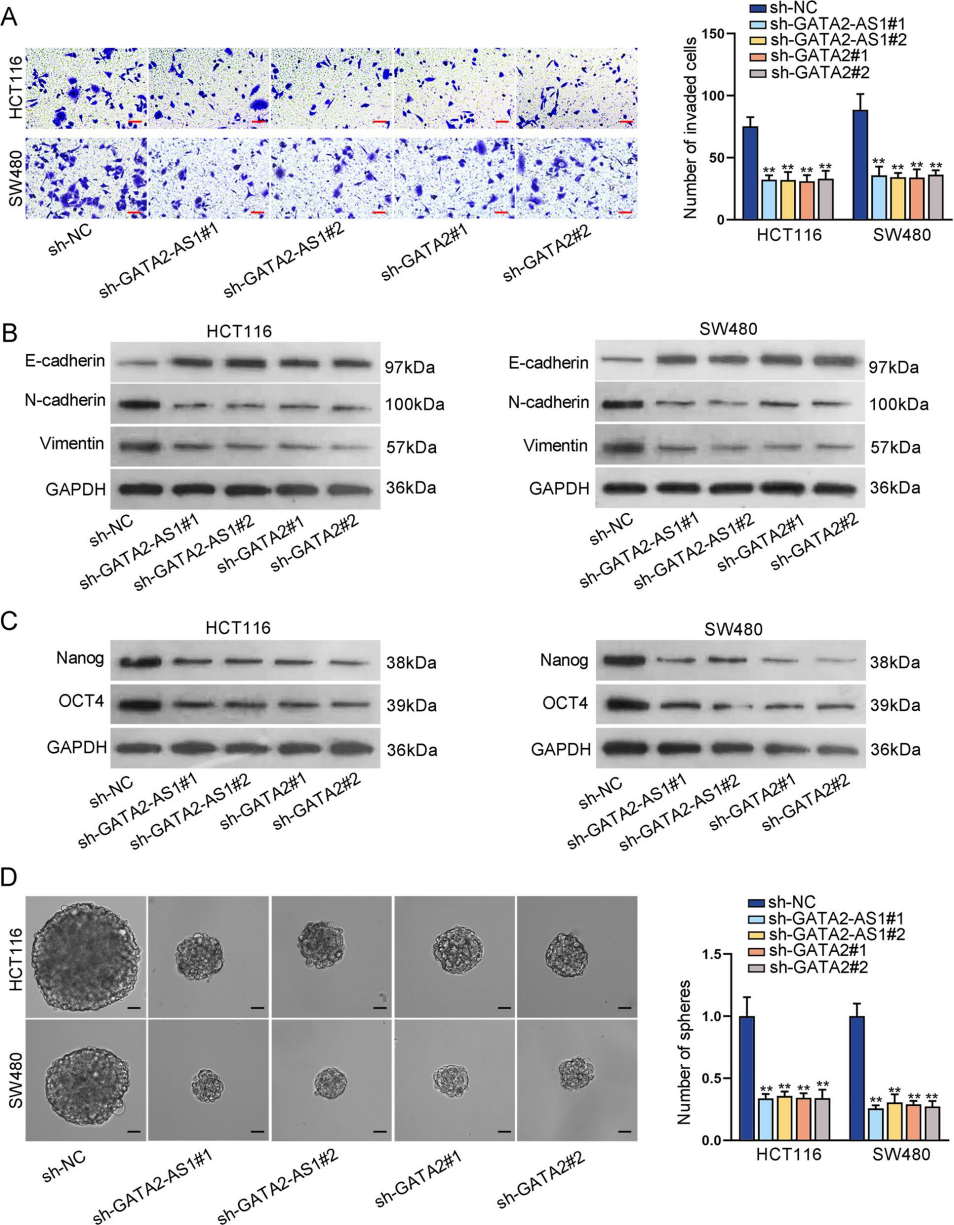
GATA2-AS1 and GATA2 promote CRC cell invasion, EMT and stemness. **A** The impacts of GATA2-AS1 or GATA2 depletion on CRC cell invasion were analyzed by transwell invasion assays. **B** The protein levels of EMT markers were measured in GATA2-AS1-shRNA or GATA2-shRNA transfected SW480 and HCT116 cells. **C**, **D** Western blot analysis and sphere formation assays measured the protein levels of stem cell markers (Nanog and OCT4) and the number and size of spheres were suppressed after GATA2-AS1 or GATA2 reduction. ^**^P < 0.01

Moreover, we investigated the impacts of GATA2-AS1 and GATA2 on CRC cell invasion, EMT and stemness. Transwell assays showed that the number of invading cells was obviously reduced after the depletion of GATA2-AS1 or GATA2 (Fig. 3A). Besides, we analyzed the protein levels of EMT markers in GATA2-AS1-inhibited or GATA2-inhibited SW480 and HCT116 cells via western blot. Down-regulation of GATA2-AS1 or GATA2 led to an obvious elevation in the protein level of epithelial marker (E-cadherin) and a distinct reduction in those of mesenchymal markers (N-cadherin and Vimentin) (Fig. 3B). In addition, the data from western blot analysis and sphere formation assay disclosed that the protein levels of stem cell markers (Nanog and OCT4) and the number and size of spheres were overtly reduced after GATA2-AS1 or GATA2 knockdown (Fig. 3C, D). All above data indicated that GATA2-AS1 and GATA2 promote CRC cell invasion, EMT and stemness.

### GATA2-AS1 recruits DDX3X to control the stability of GATA2 mRNA

**Fig. 4:**
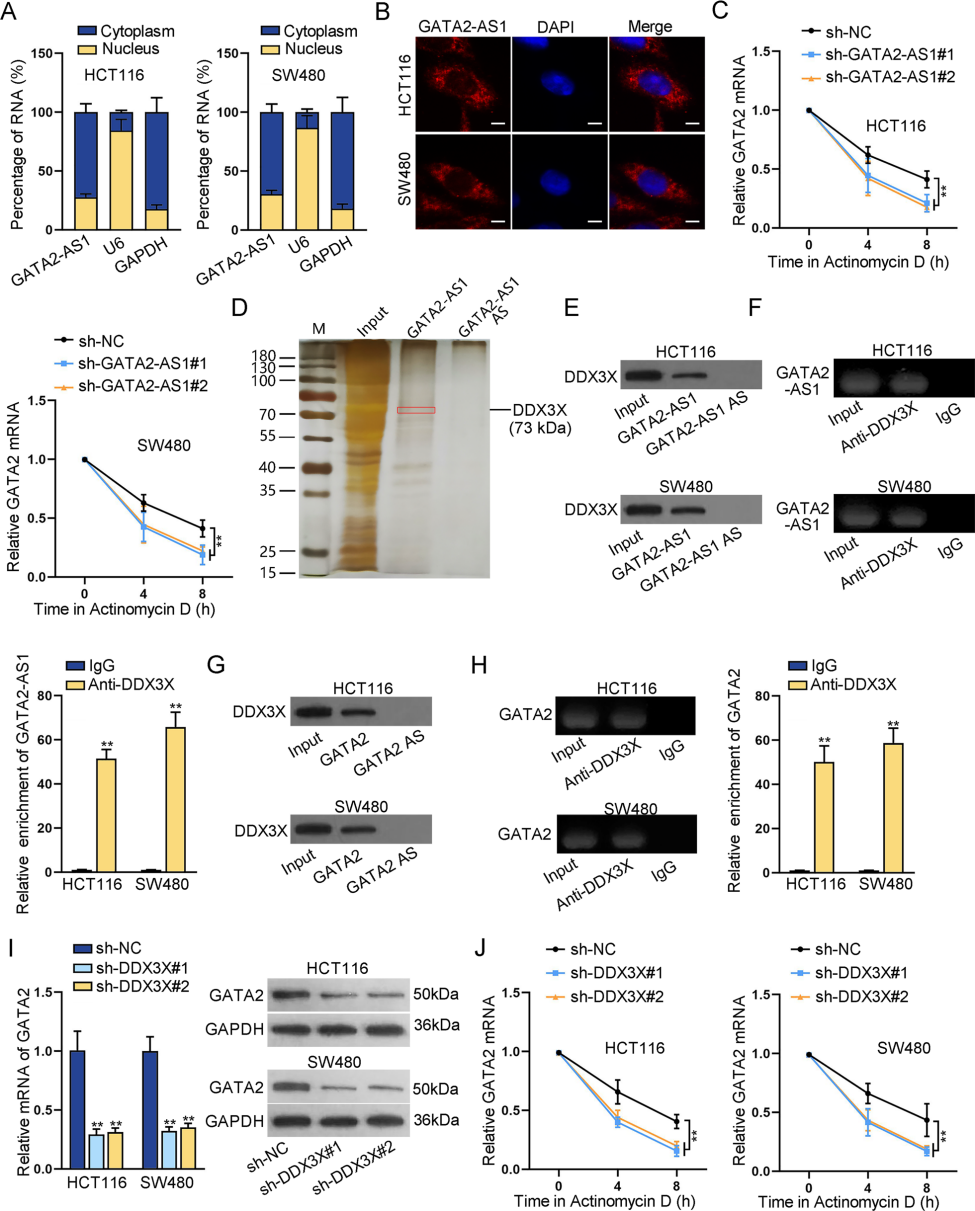
GATA2-AS1 recruits DDX3X to control the stability of GATA2 mRNA. **A**, **B** The cytoplasmic distribution of GATA2-AS1 in CRC cells was verified by subcellular fractionation and FISH assays. **C** ActD assays detected the stability of GATA2 in GATA2-AS1-shRNAs transfected SW480 and HCT116 cells. **D**, **E** RNA pull down assay showed the physical interaction between GATA2-AS1 and DDX3X. **F** The enrichment of GATA2-AS1 in Anti-DDX3X groups was detected by RIP assays. **G**, **H** RNA pull down and RIP assays detected the binding between GATA2 and DDX3X. **I** GATA2 RNA and protein levels were detected in DDX3X-ablated CRC cells. **J** ActD assays detected the stability of GATA2 in DDX3X-shRNAs transfected CRC cells. ^**^P < 0.01

Furthermore, we investigated the regulatory mechanism between GATA2-AS1 and GATA2. The subcellular distribution of lncRNA is associated with its mechanism [25]. GATA2-AS1 was verified to be primarily localized in the cytoplasm of CRC cells (Fig. 4A, B), which implied the potential of GATA2-AS1 to post-transcriptionally regulate GATA2 expression. Notably, competing endogenous RNA (ceRNA) is a common post-transcriptional regulation mechanism functioning in the development of cancers [26]. Given that, we next explored miRNAs binding with GATA2-AS1 or GATA2; potential miRNAs were predicted by ENCORI (https://rna.sysu.edu.cn/encori/). However, there was no appropriate miRNA binding to both GATA2-AS1 and GATA2 (Additional file 2: Fig. S2C), which excluded ceRNA regulation mechanism. Subsequently, ActD was used to inhibit protein synthesis in the analysis of GATA2 mRNA stability. The results showed that GATA2-AS1 silencing obviously decreased GATA2 mRNA level in ActD-treated CRC cells (Fig. 4C). LncRNAs could recruit some proteins to stabilize mRNA [27]. Hence we carried out RNA pull down assay to explore potential RBPs of GATA2-AS1. A ~ 73 kDa protein was pulled down by GATA2-AS1 and identified as DDX3X via mass spectrometry (Fig. 4D). RNA pull down and RIP assays further confirmed the interaction between GATA2-AS1 and DDX3X (Fig. 4E, F). Also, it was found that DDX3X protein was pulled down by GATA2 (Fig. 4G); GATA2 was highly enriched in anti-DDX3X bound precipitates (Fig. 4H), indicating the binding between GATA2 and DDX3X. We next investigated the impact of DDX3X on the stability of GATA2 mRNA in CRC cells. We silenced the expression of DDX3X by transfection of sh-DDX3X#1/2 (Additional file 2: Fig. S2D). GATA2 mRNA and protein levels were significantly reduced after DDX3X depletion (Fig. 4I). Therefore, we hypothesized whether GATA2-AS1 regulates DDX3X to affect the expression of GATA2. GATA2-AS1 deletion had no marked effect on DDX3X expression at mRNA and protein levels (Additional file 2: Fig. S2E, F). Whereupon, we speculated that GATA2-AS1 might recruit DDX3X to regulate the stability of GATA2. GATA2 mRNA level was significantly decreased in ActD-treated cells after DDX3X silencing (Fig. 4J), indicating that DDX3X promotes the stability of GATA2 mRNA. All these data revealed that GATA2-AS1 recruits DDX3X to regulate the stability of GATA2 mRNA, affecting the expression of GATA2.

### GATA2 transcriptionally activates GATA2-AS1 expression

**Fig. 5:**
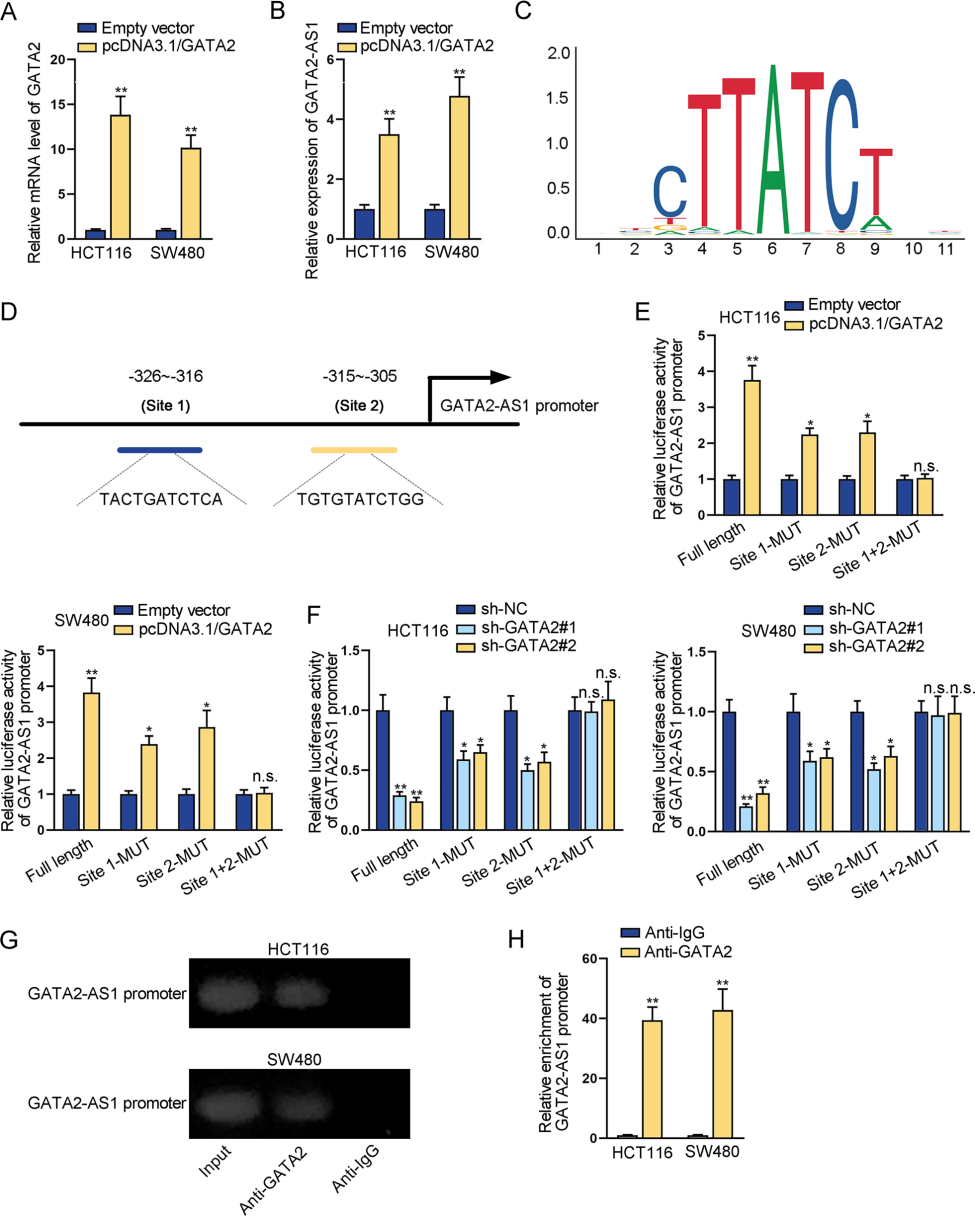
GATA2 transcriptionally activates GATA2-AS1 expression. **A** RT-qPCR verified gene overexpression efficiency of GATA2 in CRC cells. **B** RT-qPCR was used to detect GATA2-AS1 expression in GATA2-overexpressing cells. **C**, **D** JASPAR database showed two potential GATA2-binding sites on GATA2-AS1 promoter. **E**, **F** Luciferase reporter assays detected the luciferase activity of GATA2-AS1 promoter at Full length, Site 1-MUT, Site 2-MUT and Site 1 + 2-MUT in CRC cells transfected with pcDNA3.1/GATA2 or shRNAs targeting GATA2. **G**, **H** ChIP assays detected the binding between GATA2-AS1 promoter and GATA2. ^*^P < 0.05, ^**^P < 0.01, n.s.: no significance

Based on the analysis of UCSC database (http://genome.ucsc.edu/), we obtained a list of putative transcription factors of GATA2-AS1 (Additional file 3: Fig. S3). Notably, GATA2 was one of the listed transcription factors. Thereby, we probed into the impact of GATA2 as a transcription factor on GATA2-AS1 expression at the transcriptional level. GATA2 was successfully overexpressed via transfection of pcDNA3.1/GATA2 (Fig. 5A). RT-qPCR showed that GATA2-AS1 expression was obviously elevated when GATA2 was overexpressed (Fig. 5B). With the help of JASPAR (http://jaspar.genereg.net/), we found two potential GATA2-binding sites on the sequence of GATA2-AS1 promoter (Fig. 5C, D). Site 1 or/and Site 2 mutations were performed for subsequent luciferase reporter assays. The luciferase activity of full length GATA2-AS1 promoter (Full length) or mutant GATA2-AS1 promoter (Site 1-MUT and Site 2-MUT) was significantly enhanced by GATA2 overexpression, while that of mutant GATA2-AS1 promoter (Site 1 + 2-MUT) was barely affected (Fig. 5E). Also, GATA2 silencing caused a decrease in the luciferase activity of GATA2-AS1 promoter (Full length) and mutant GATA2-AS1 promoter (Site 1-MUT and Site 2-MUT), but caused no marked change in that of mutant one (Site 1 + 2-MUT) (Fig. 5F). The above data indicated that both Site 1 and Site 2 are GATA2-binding sites in GATA2-AS1 promoter. Additionally, ChIP assay further confirmed the binding between GATA2-AS1 promoter and GATA2, as GATA2-AS1 promoter was overtly abundant in anti-GATA2 bound precipitates (Fig. 5G, H). Taken together, GATA2 transcriptionally activates GATA2-AS1 expression.

### GATA2-AS1 affects CRC cell proliferation and apoptosis via regulating GATA2 expression

**Fig. 6:**
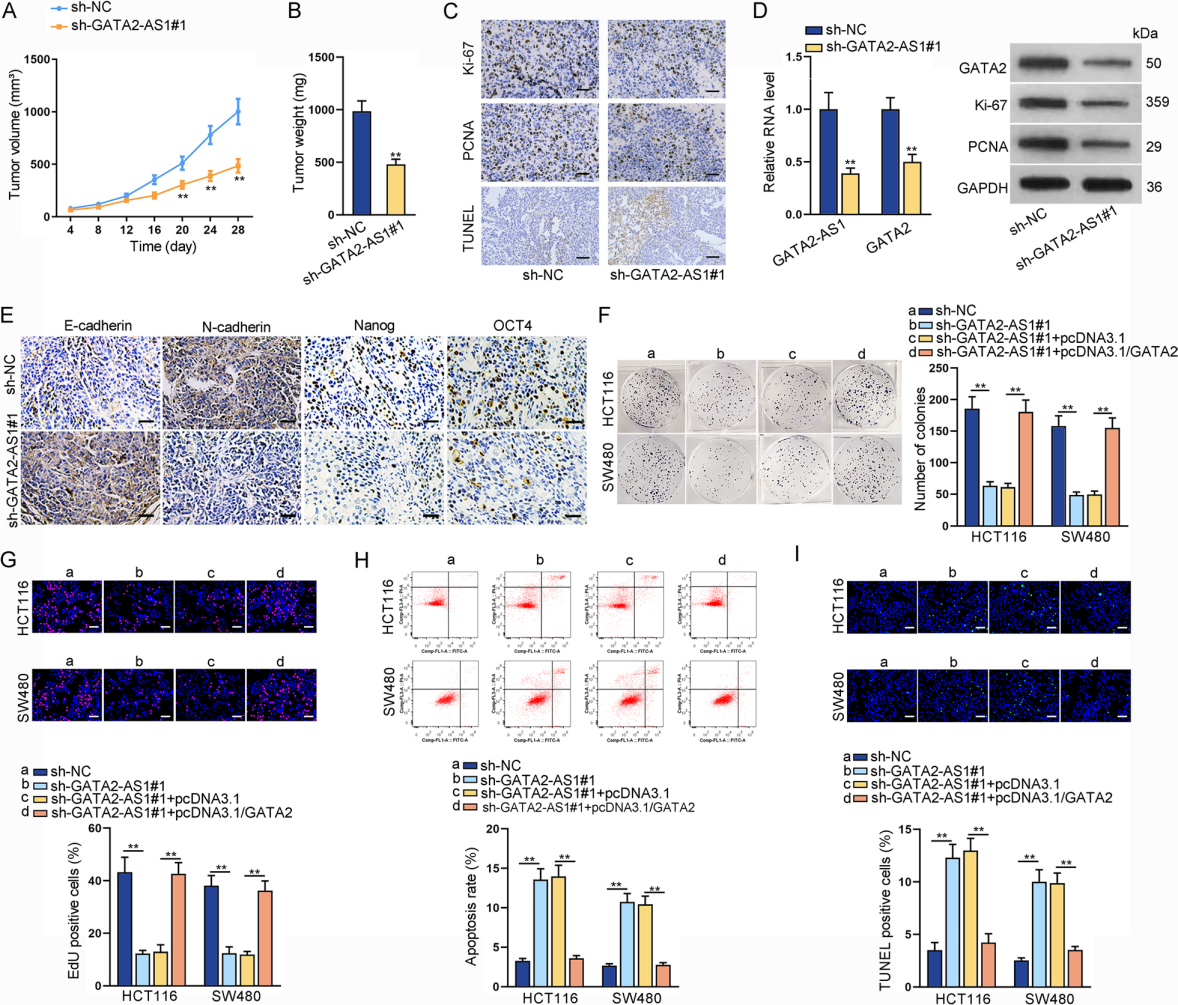
GATA2-AS1 affects CRC cell proliferation and apoptosis via regulating GATA2 expression. In vivo experiment using stably transfected HCT116 cells was conducted. **A**, **B** Tumor volume and weight of mouse xenografts were measured. **C** IHC staining was used to detect the expression of Ki-67 and PCNA in xenograft tissues; TUNEL staining detected the apoptosis of tumor tissues from mouse xenografts. **D** The expression of GATA2-AS1 and GATA2 and protein levels of GATA2, Ki-67 and PCNA were detected in tumor tissues from mouse xenografts. **E** IHC staining detected the expression of E-cadherin, N-cadherin, Nanog and OCT4 in tumor tissues from mouse xenografts. Rescue experiments were conducted in HCT116 and SW480 cells treated with different transfections. **F**, **G** The proliferation of CRC cells in different groups was evaluated. **H**, **I** The apoptosis of CRC cells in different groups was assessed. ^**^P < 0.01

Besides in vitro assays, we established xenograft tumor models to verify the role of GATA2-AS1 in CRC tumorigenesis. Stably sh-GATA2-AS1#1 or sh-NC transfected CRC cells were subcutaneously injected into nude mice to construct mouse xenograft models. The tumor volume and tumor weight were significantly declined when GATA2-AS1 was knocked down (Fig. 6A, B; Additional file 4: Fig. S4A, B). Besides, IHC staining revealed that the expression of proliferative markers (Ki-67 and PCNA) was significantly inhibited in sh-GATA2-AS1#1 group compared to sh-NC group; TUNEL staining revealed that GATA2-AS1 inhibition promotes the apoptosis of xenograft tumors (Fig. 6C; Additional file 4: Fig. S4C). Moreover, the RNA levels of GATA2-AS1 and GATA2 were decreased in GATA2-AS1-inhibited group; the protein levels of GATA2, Ki-67 and PCNA were reduced when GATA2-AS1 was silenced (Fig. 6D; Additional file 4: Fig. S4D). Meanwhile, EMT and stemness were also repressed when GATA2-AS1 was knocked down (Fig. 6E; Additional file 4: Fig. S4E). All these data suggested that knockdown of GATA2-AS1 inhibits tumor growth, EMT and stemness in CRC.

Additionally, we performed rescue experiments to validate the impact of GATA2-AS1-GATA2 interaction on CRC cell proliferation and apoptosis. GATA2 overexpression reversed the inhibited proliferation ability of GATA2-AS1-silenced CRC cells (Fig. 6F, G). Besides, the increased apoptosis caused by GATA2-AS1 depletion was counteracted by GATA2 overexpression (Fig. 6H, I). In sum, GATA2-AS1 regulates CRC cell proliferation and apoptosis through the modulation of GATA2 expression.

### GATA2-AS1 facilitates CRC cell invasion, EMT and stemness via modulating GATA2 expression

**Fig. 7:**
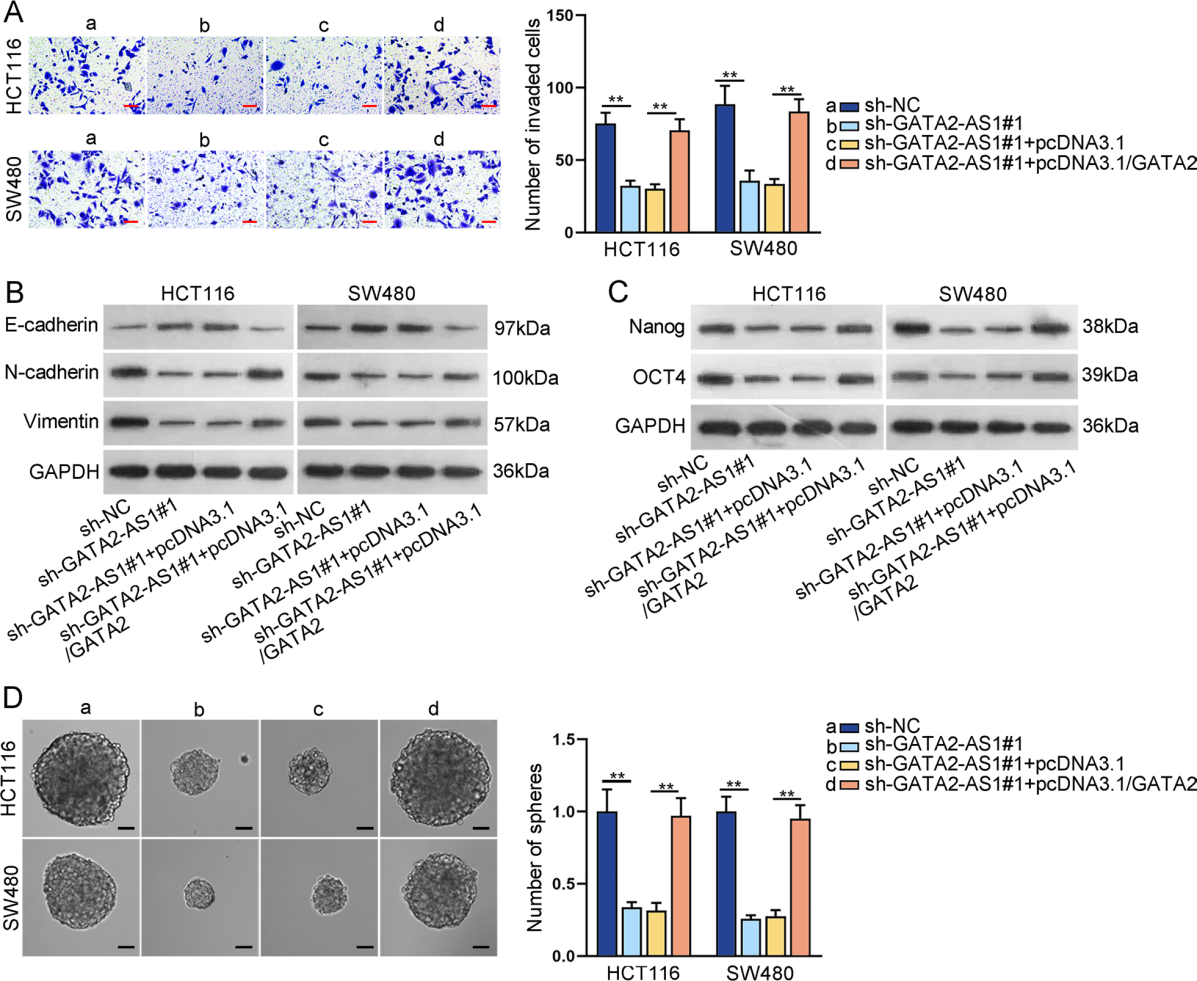
GATA2-AS1 facilitates CRC cell migration, EMT and stemness via modulating GATA2 expression. Rescue experiments were conducted in HCT116 and SW480 cells transfected with indicated plasmids. **A** Transwell assays were conducted to detect the migration ability of CRC cells. **B**, **C** Western blot was used to measure the protein levels of EMT markers (E-cadherin, N-cadherin and Vimentin) and stem cell markers (Nanog and OCT4) in CRC cells. **D** Sphere formation assay was done to determine the stemness of CRC cells. ^**^P < 0.01

Moreover, we conducted rescue experiments to verify the impacts of GATA2-AS1-GATA2 interaction on CRC cell invasion, EMT and stemness. As evidenced by transwell assays, GATA2 overexpression countervailed the repression of GATA2-AS1 depletion on CRC cell migration (Fig. 7A). Western blot revealed that the inhibition of GATA2-AS1 silencing on EMT was offset by GATA2 overexpression (Fig. 7B). In addition, the declined protein levels of Nanog and OCT4 and the reduced number and size of spheres in GATA2-AS1-silenced CRC cells were altered by up-regulation of GATA2 (Fig. 7C, D). Collectively, GATA2-AS1 facilitates CRC cell migration, EMT and stemness via up-regulating GATA2 expression.

## Discussion

Previous studies have highlighted the critical role of biological and molecular markers in the diagnosis, prognosis, and treatment of malignant tumors and CRC [28–31]. Here, the present study demonstrated a novel regulation mechanism that GATA2-activated GATA2-AS1 up-regulation facilitates CRC cell proliferation, invasion, EMT and stemness through interaction with RBP DDX3X, which might be helpful to explore new targets for CRC treatment (Additional file 5: Fig. S5).

LncRNAs can modulate the expression of nearby genes to involve in cancer development. As reported previously, MAPKAPK5-AS1 facilitates CRC progression via sponging let-7f-1-3p to cis-regulate its nearby gene MK5 [32]. TRPM2-AS increases CRC cell proliferation via TRPM2; TRPM2-AS directly interacts with TAF15 protein to maintain the mRNA stability of TRPM2 [17]. Consistent with these reports, the present study explored the interaction of GATA2-AS1 and its nearby gene GATA2 in CRC. GATA2 has been identified to be high-expressed in CRC and its high expression is associated with recurrence of CRC, which suggests that GATA2 is a useful prognostic indicator of CRC treatment [33]. In our study, we verified that GATA2-AS1 and GATA2 exhibited high expression levels in CRC cell lines. Functionally, silencing of GATA2-AS1 and GATA2 represses CRC cell proliferation, invasion and stemness and induces cell apoptosis. EMT is an important biological process and plays crucial roles in regulating embryonic development, chronic inflammation, tissue reconstruction as well as tumor metastasis [34]. During EMT, cells are involved in the loss of epithelial characteristics such as epithelial marker (E-cadherin) and the acquisition of mesenchymal phenotype such as mesenchymal markers (N-cadherin and Vimentin) [35]. Increasing reports have demonstrated that lncRNAs positively regulate the EMT process in CRC [36]. Similarly, our study confirmed that depletion of GATA2-AS1 or GATA2 obviously inhibits EMT process in CRC. All above data implied the cancer-promoting role of GATA2-AS1 in CRC, which unveiled that GATA2-AS1 might be a novel target for CRC treatment.

It has been reported that GATA2-AS1 regulates GATA2 to impair non-small cell lung cancer cell proliferation via interacting with GATA1 protein at GATA2 promoter region and then blocks its transcription [22]. Unlike with that report, our study found that GATA2-AS1 positively regulates GATA2 expression at the post-transcriptional level. In recent years, ceRNA networks generated by lncRNA-miRNA-mRNA interactions have been shown to exert function in various biological processes in CRC [37, 38]. However, our study found that there was no common miRNA binding with both GATA2-AS1 and GATA2 through database analysis. Thereby, we turned to explore RBP mechanism of GATA2-AS1-mediated regulation in CRC. Previous study has suggested that DDX3X serves as a RBP to regulate the half-life of Zc3h12a mRNA [39]. In addition, DDX3X has been suggested to exhibit oncogenic function in multiple cancers, such as breast cancer [40] and glioma [41]. Of note, DDX3X, highly expressed in CRC, is regarded as a promising therapeutic target for CRC [42]. In this research, we further validated that GATA2-AS1 interacts with DDX3X to control the stability of GATA2 mRNA regulating its expression.

Moreover, GATA transcription factors are necessary in mammalian cell lineage determination and play a significant role in cancer development [43]. GATA2 is a member of GATA family and serves as a transcription factor expressed in early progenitor cells; GATA2 is implicated in modulating the fate of hematopoietic stem cells and progenitor cells [44]. GATA2 interacts with androgen receptor to modulate gene transcription in prostate cancer cells [45]. In this study, we found that GATA2 combines with GATA2-AS1 promoter to enhance GATA2-AS1 expression. In addition, we validated the oncogenic effect of GATA2-AS1 on tumor growth, EMT and stemness through in vivo experiments. Furthermore, this study further validated that GATA2-AS1 promotes CRC cell proliferation, invasion, EMT and stemness via up-regulating GATA2 expression.

## Conclusion

In conclusion, our study identifies a feedback loop between GATA2-AS1 and GATA2. GATA2 is able to interact with the promoter of GATA2-AS1 inducing its expression. In turn, GATA2-AS1 recruits DDX3X on the 3’UTR of GATA2 regulating its expression post-transcriptionally. This mechanism promotes the progression of CRC, controlling proliferation, invasion, EMT processes and stemness, and might supply a novel sight for CRC treatment. Intriguingly, GATA2 is a downstream transcription factor of the p38 and JNK pathways in CRC [46], and our study will further investigate the interaction of GATA2 and signaling pathways.

## Supplementary Information

**Additional file 1: Figure S1. RNA-seq of GATA2-AS1 in 27 different normal tissues.**

**Additional file 2: Figure S2. Knockdown or overexpression efficiency of plasmids.** A RT-qPCR verified the interference efficiency of sh-GATA2-AS1#1 and sh-GATA2-AS1#2 in transfected CRC cells. B The interference efficiency of sh-GATA2#1 and sh-GATA2#2 in transfected CRC cells was elucidated. C ENCORI predicted miRNAs combining with GATA2-AS1 or GATA2. D Gene deletion efficiency of DDX3X in CRC cells was validated. E-F DDX3X level was detected in GATA2-AS1-silenced CRC cells. ^**^P<0.01, n.s. indicated no significance.

**Additional file 3: Figure S3. Transcription factors of GATA2-AS1 predicted by UCSC database.**

**Additional file 4: Figure S4. In vivo experiment using stably transfected SW480 cells.** A-B Tumor volume and weight of mouse xenografts were measured. C IHC staining of Ki-67 and PCNA expression in mouse xenograft tissues; the apoptosis of tumor tissues from mouse xenografts was detected by TUNEL. D RT-qPCR was used to detect GATA2-AS1 and GATA2 levels, and western blot to analyze GATA2, Ki-67 and PCNA protein levels in mouse xenograft tissues. E IHC staining of E-cadherin, N-cadherin, Nanog and OCT4 expression in tumor tissues from mouse xenografts was shown. ^**^P<0.01.

**Additional file 5: Figure S5. Schematic showing the regulatory mechanism of GATA2-AS1 in CRC cells.**

## Data Availability

Not applicable.

## Abbreviations

lncRNAs: Long non-coding RNAs
CRC: Colorectal cancer
GATA2-AS1: GATA binding protein 2 antisense RNA 1
GATA2: GATA binding protein 2
DDX3X: DEAD-box helicase 3 X-linked
RBPs: RNA-binding proteins
EMT: Epithelial-mesenchymal transition
RT-qPCR: Quantitative real-time polymerase chain reaction
RIP: RNA-binding protein immunoprecipitation
ChIP: Chromatin immunoprecipitation
TUNEL: Terminal-deoxynucleoitidyl Transferase Mediated Nick End labeling
FISH: Fluorescent in situ hybridization
IHC: Immunohistochemical
GAPDH: Glyceraldehyde-3-phosphate dehydrogenase
SD: Standard deviation
EdU: 5-Ethynyl-2’-deoxyuridine
