# Supplementary figures and images for: A feedback loop between GATA2-AS1 and GATA2 promotes colorectal cancer cell proliferation, invasion, epithelial-mesenchymal transition and stemness via recruiting DDX3X
Source: J Transl Med. 2022 Jun 25;20:287. doi: 10.1186/s12967-022-03483-8 (PMC9233859; doi:10.1186/s12967-022-03483-8)

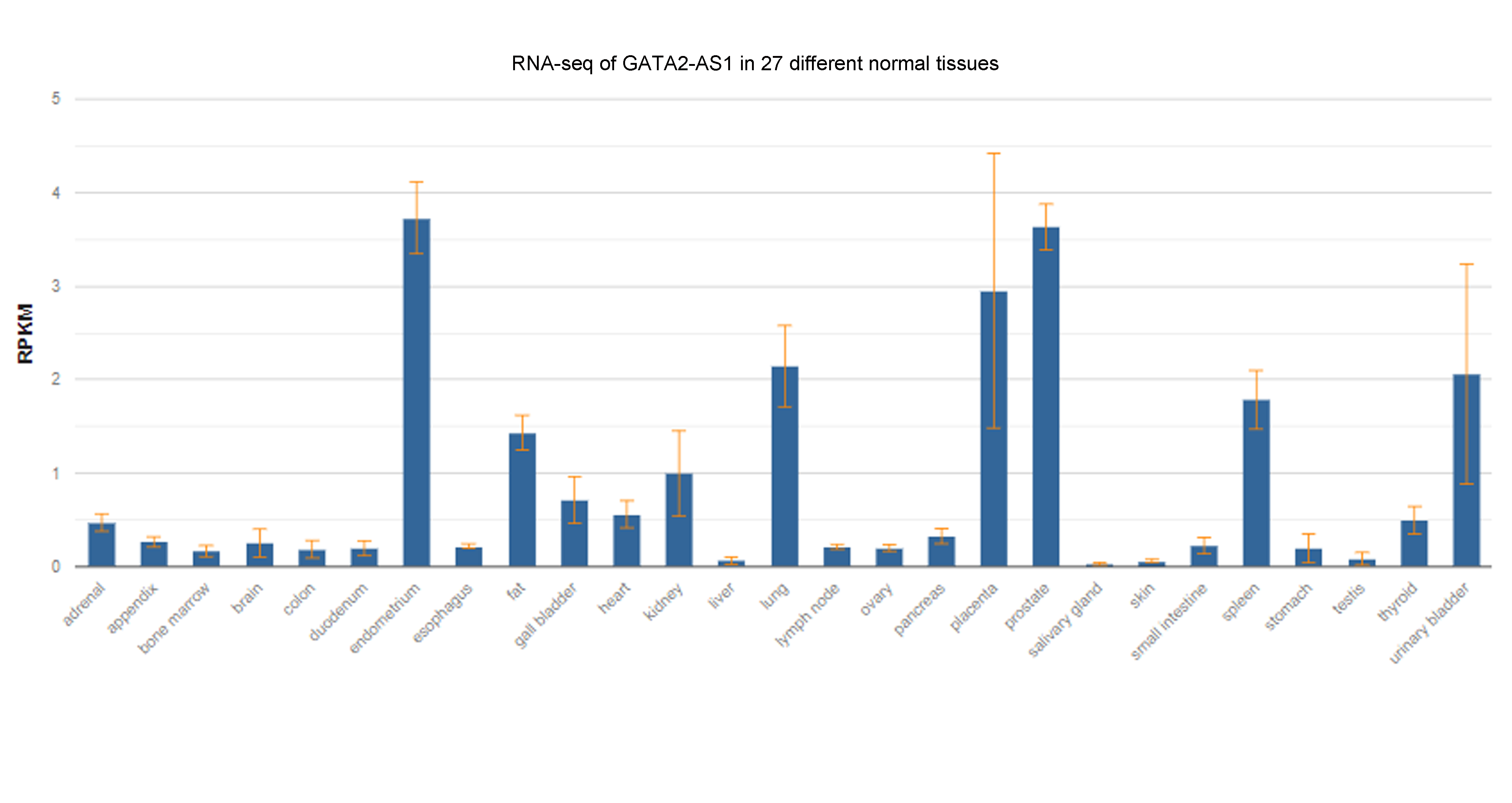

Supplement: Supplementary file 1 — Additional file 1: Figure S1. RNA-seq of GATA2-AS1 in 27 different normal tissues. [file 12967_2022_3483_MOESM1_ESM.tif]

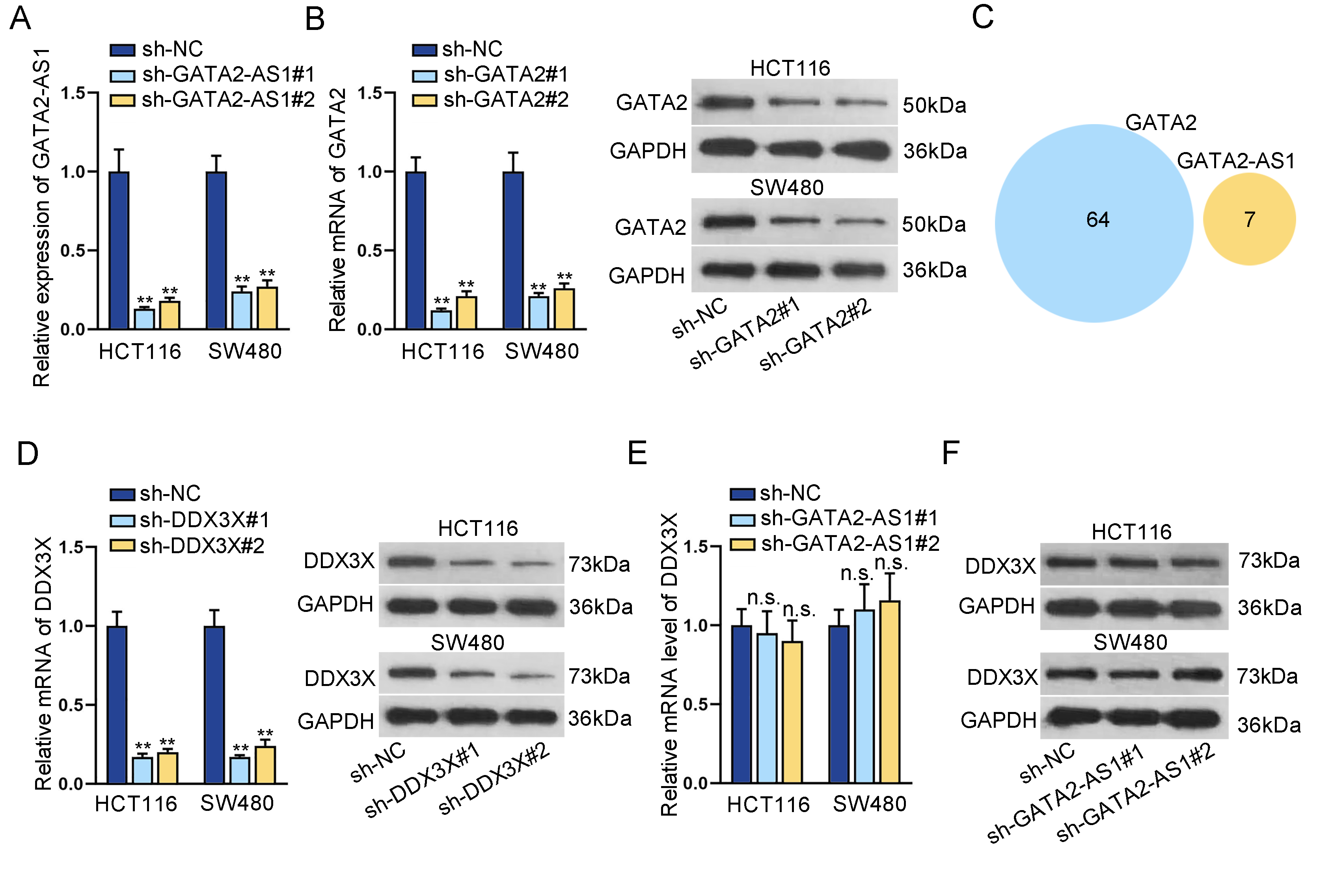

Supplement: Supplementary file 2 — Additional file 2: Figure S2. Knockdown or overexpression efficiency of plasmids. A RT-qPCR verified the interference efficiency of sh-GATA2-AS1#1 and sh-GATA2-AS1#2 in transfected CRC cells. B The interference efficiency of sh-GATA2#1 and sh-GATA2#2 in transfected CRC cells was elucidated. C ENCORI predicted miRNAs combining with GATA2-AS1 or GATA2. D Gene deletion efficiency of DDX3X in CRC cells was validated. E-F DDX3X level was detected in GATA2-AS1-silenced CRC cells. **P<0.01, n.s. indicated no significance. [file 12967_2022_3483_MOESM2_ESM.tif]

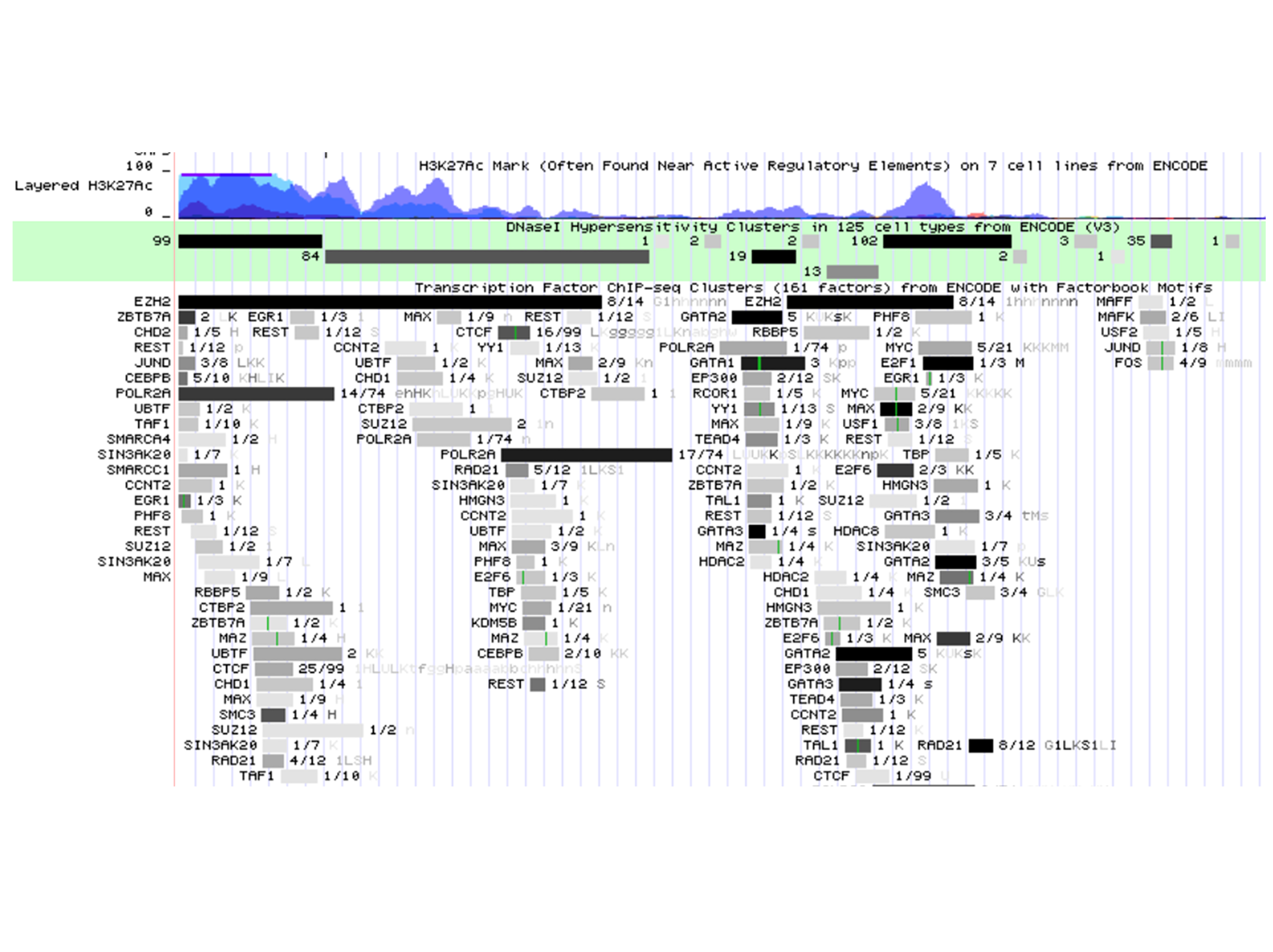

Supplement: Supplementary file 3 — Additional file 3: Figure S3. Transcription factors of GATA2-AS1 predicted by UCSC database. [file 12967_2022_3483_MOESM3_ESM.tif]

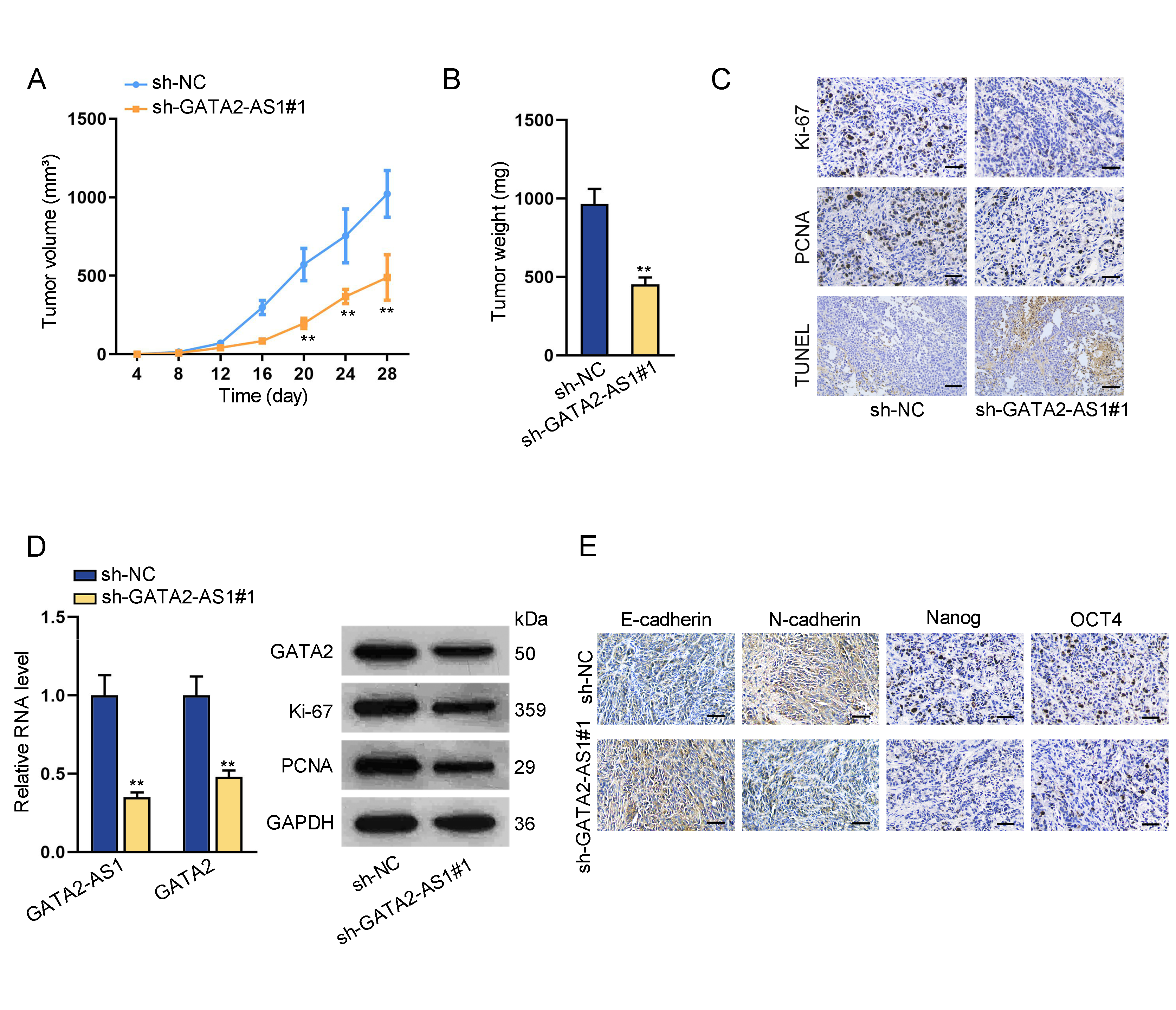

Supplement: Supplementary file 4 — Additional file 4: Figure S4. In vivo experiment using stably transfected SW480 cells. A-B Tumor volume and weight of mouse xenografts were measured. C IHC staining of Ki-67 and PCNA expression in mouse xenograft tissues; the apoptosis of tumor tissues from mouse xenografts was detected by TUNEL. D RT-qPCR was used to detect GATA2-AS1 and GATA2 levels, and western blot to analyze GATA2, Ki-67 and PCNA protein levels in mouse xenograft tissues. E IHC staining of E-cadherin, N-cadherin, Nanog and OCT4 expression in tumor tissues from mouse xenografts was shown. **P<0.01. [file 12967_2022_3483_MOESM4_ESM.tif]

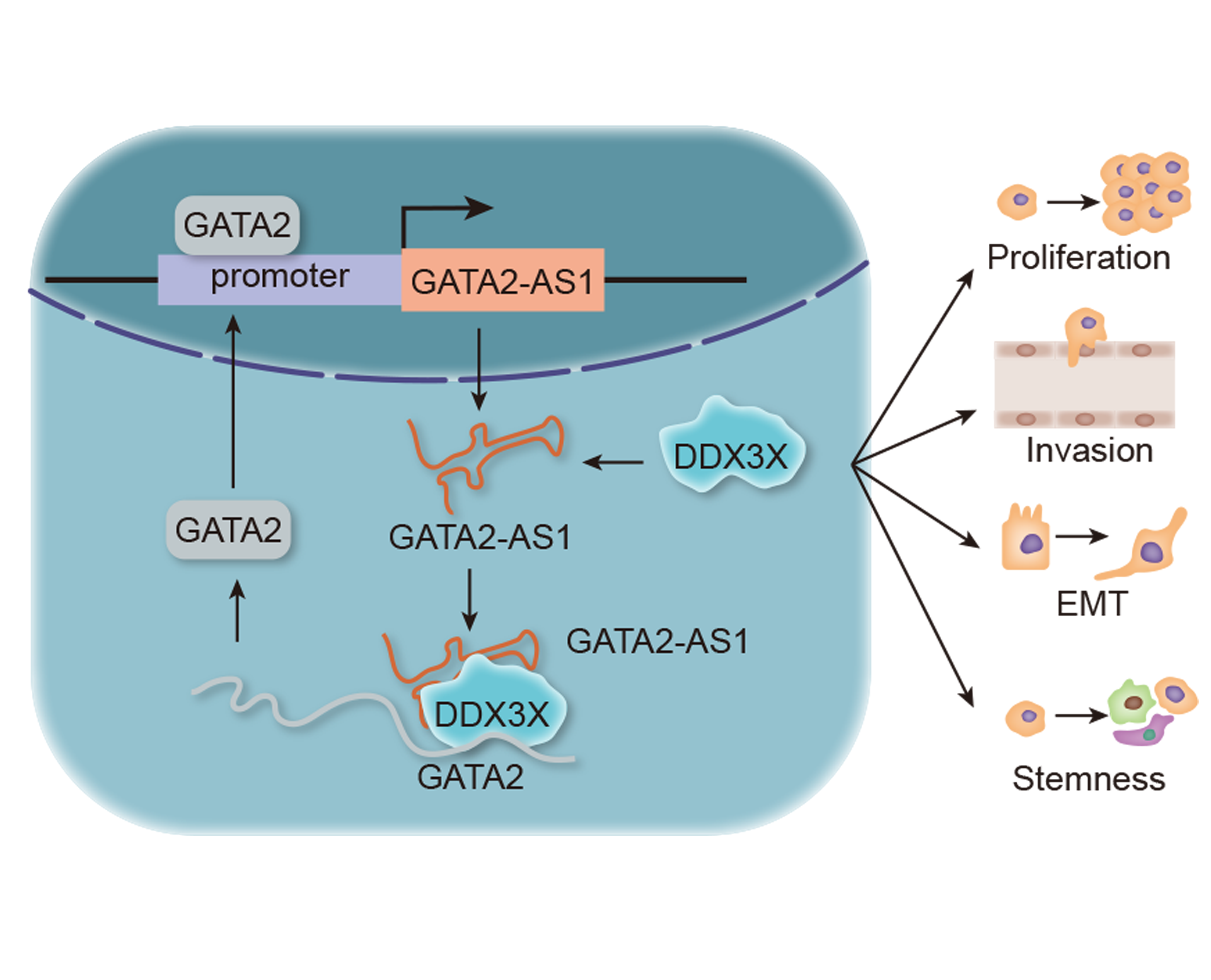

Supplement: Supplementary file 5 — Additional file 5: Figure S5. Schematic showing the regulatory mechanism of GATA2-AS1 in CRC cells. [file 12967_2022_3483_MOESM5_ESM.tif]
